# Use of autologous fascia lata graft to repair a complex corneal ulcer in a mare

**DOI:** 10.1186/s13620-020-00160-4

**Published:** 2020-05-05

**Authors:** Marcos Lores, Peter Rakestraw, Mieke De Rijck, Thomas Yarbrough

**Affiliations:** 1 Sharjah Equine Hospital, P.O. Box 29858, Sharjah, United Arab Emirates; 2 Dubai Equine Hospital, 2 St N 22A, Dubai, United Arab Emirates

**Keywords:** Ulcerative keratitis, Stromal keratomalacia, Keratomycosis, Corneal aspergillosis, Conjunctival graft, Fascia lata

## Abstract

**Background:**

Application of an autogenous fascia lata graft in the treatment of keratomalacia in the horse has not been reported. The present case describes the use of an autologous fascia lata graft to surgically treat a complicated corneal ulcer in a horse.

**Case presentation:**

A 12-year-old Arabian mare was admitted to Sharjah Equine Hospital with a history of right eye ulcerative keratitis of unknown duration. Following a week of aggressive medical treatment, the condition deteriorated and a keratectomy and pedicle conjunctival graft were performed. A week later, the conjunctival graft partially dehisced and the ulcer continued to degrade.

In attempting to preserve the integrity of the globe a second grafting procedure was performed when a segment of fascia lata harvested from the ipsilateral hind leg was grafted onto the corneal lesion.

Response to surgery was positive and the mare was discharged from the hospital on Day 31 post diagnosis (17 days after the second surgery) with a comfortable eye.

Follow-up12 months later revealed a comfortable but only partially visual eye due to corneal scarring.

**Conclusion:**

Although the graft actually failed from the point of view of tissue integration, fascia lata appears to be a readily available, effective and affordable autografting material and we believe this technique has potential for short term physical and structural support of severe malacic or other complex corneal ulcers.

## Background

Corneal ulceration occurs commonly in equids [1] usually as a consequence of trauma. Many of these corneal lesions are superficial and resolve with medical therapy [2]. However, when such treatment is unsuccessful, progressive deepening of the ulcer, corneal malacia (melting) and necrosis can lead to descemetocele formation and perforation of the cornea [2]. Therefore, any corneal defect deep enough to threaten corneal integrity (involvement of half of the corneal thickness or more) warrants surgical repair by corneal reconstruction/grafting [2]. Several biomaterials have been described for use in corneal grafting including allogenous and autogenous cornea, conjunctiva, amniotic membrane, porcine urinary bladder extracellular matrix grafts, porcine small intestine submucosa and bovine pericardium [3–10].

The goal of corneal grafting is to preserve the integrity of the globe, provide tectonic support, reduce ocular pain, optimize the visual outcome and minimize scarring. The ideal biomaterial for such corneal reconstruction would provide optical clarity, provide resistance to endogenous corneal proteases, and promote epithelial growth [3, 11].

Although the application of an autogenous fascia lata graft to repair a traumatic scleral defect has been reported in the dog [12], its application in the treatment of keratomalacia in the horse has not previously been reported. The present case describes the use of an autologous fascia lata graft to surgically treat a complicated corneal ulcer in a horse. In addition to be easily harvested and inexpensive, the fascial material proved to be substantially thicker and stronger than the traditional conjunctival flap, or commonly used amnion, and it was therefore expected to show greater tectonic properties.

## Case presentation

A 12-year-old, 425-kg Arabian broodmare was at pasture when right eye blepharospasm and a focal corneal opacity were noted by the keeper, leading to referral to Sharjah Equine Hospital for evaluation and treatment.

On presentation, general physical examination was unremarkable. Moderate right eye blepharospasm, mild epiphora with mucopurulent ocular discharge, an obvious corneal lesion and conjunctival hyperemia were noted to be present.

The findings of the ophthalmic examination included right eye normal ocular adnexa, normal menace responses, dazzle and palpebral reflexes in both eyes. Consensual and direct pupillary light reflexes were elicited from the left eye but not accurately evaluated on the right eye due to extent of the corneal disease and intraocular inflammation. Following sedation with xylazine (Xylazyl, Troy laboratories PTY Limited, Glendenning, Australia) (0.5 mg/kg, IV), subcutaneous auriculopalpebral and supraorbital nerve blocks using lidocaine hydrochloride 2% (Lignocaine, Troy laboratories PTY Limited, Glendenning, Australia) (0.5–1 mL) were used to facilitate ocular examination and before any topical medication, the corneal lesion was swabbed for aerobic bacterial and fungal culture. Fluorescein staining was then performed (Ful-glo, Akron Inc., Buffalo Grove, IL, USA) and the eye was closely evaluated under magnification (Portable split-lamp biomicroscopy, Kowa SL-14). A 10-mm-diameter lesion, which retained fluorescein stain, was evident in the central aspect of the cornea. This defect was seen as a peripheral epithelial lesion surrounding the malacic stroma (keratomalacia) with loss of the anterior quarter of the stroma. A shallow furrow was present at the border of the infected and non-infected cornea. There was a 7 mm long, peripheral (360°) vascular fringe or neovascularization from the limbus indicating the chronic nature of the lesion, and the cornea surrounding it was diffusely oedematous. Moderate aqueous flare, but no hypopyon, was present in the anterior chamber. It was not possible to exam the right eye fundus due to the extent of the corneal disease and intraocular inflammation. No left eye anterior segment or fundus abnormalities were seen. A Schirmer tear test (STT) was within normal limits [13]. Tonometry equipment was not available to assess intraocular pressures (IOP). After topical application of proparacaine hydrochloride ophthalmic solution (Alcaine, Alcon-Couvreur, Puurs, Belgium), the surface of the ulcer was easily scraped using the blunt edge of a stainless steel No.10 scalpel blade for exfoliative cytology.

The cytological findings revealed severe neutrophilic inflammation and mycotic keratitis, with degenerative neutrophils, epithelial cells and fungal hyphae. No intracellular bacteria were visible. The complete blood count and serum biochemistry findings were within normal limits. At 24 and 48 h, the bacterial and fungal cultures showed no growth, but at 1 week the fungal culture yielded *Aspergillus spp*. No sensitivity testing was performed.

### Treatment

A subpalpebral lavage system (Mila International, INC) was placed in the superior conjunctival fornix to facilitate the administration of medicines. Prior to acquiring the culture results 0.2 ml each of voriconazole drops (Vozole, Aurolab, Madurai, India) (q 4 h), ciprofloxacin drops (Opticin, Jamjoom Pharma, Jeddah, Saudi Arabia) (q 4 h), 1% atropine drops (Apitropin, Amman Pharmaceutical Industries Co. Sahab, Jordan) (q 12 h) and autologous serum were instilled into the lavage system (q 2 h). Systemic treatment included flunixin meglumine (Finadyne, Schering-Plough Sante Animale, La Grindoliere, France) (1.1 mg/kg, IV, q 12 h), and omeprazole (Gastropel, Randlab Australia PTY LTD, Chipping Norton, Australia) (1.0 mg/kg, PO, q 24 h). All the topical medications were delivered via a subpalpebral lavage system using 0.2 ml of the medication solutions followed by 3 ml of air to flush the tubing.

**Fig. 1:**
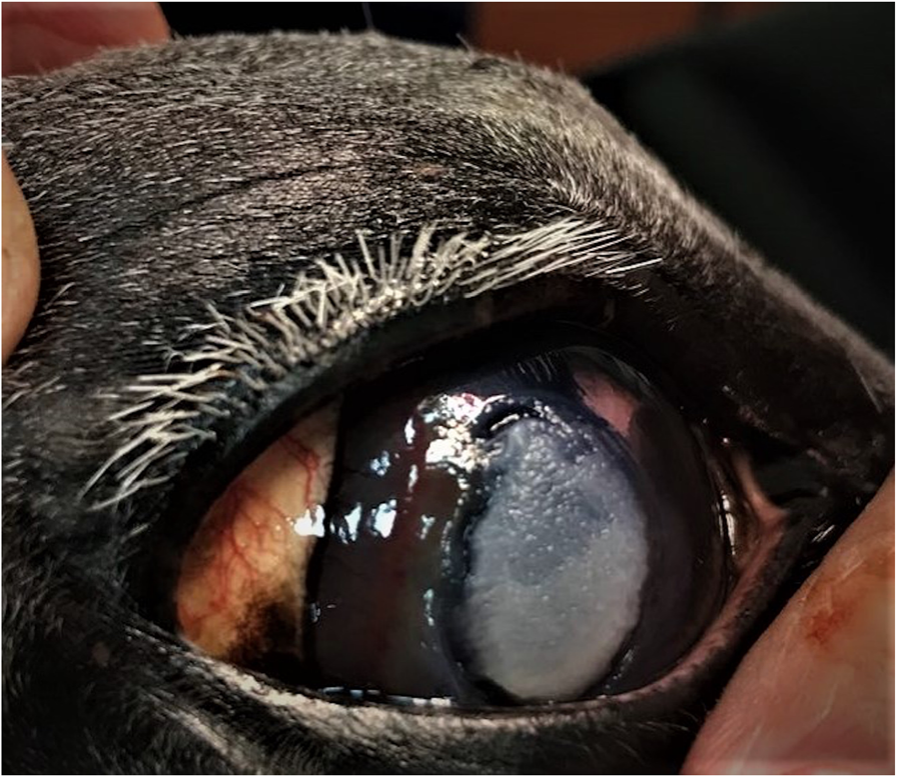
Illustrates the corneal lesion 7 days after hospitalization. Notice the dry, white, caseous exudate and the sharply defined furrow at the border of the infected and non-infected cornea

Despite this intensive therapy, the ulcer continued to worsen so that by Day 7 after admission it had degraded into a 25 mm diameter cornea lesion with the loss of approximately 60% of the stroma at the deepest aspect, and characterized by a dry, white, caseous exudate (“cake-frosting” appearance) typical of mycotic infections [14] with a sharply defined furrow at the border of the infected and non-infected cornea (Fig. 1). Due to the clear progression of the problem despite the aggressive medical therapy, surgical intervention was then recommended, and a conjunctival pedicle graft was performed.

**Fig. 2:**
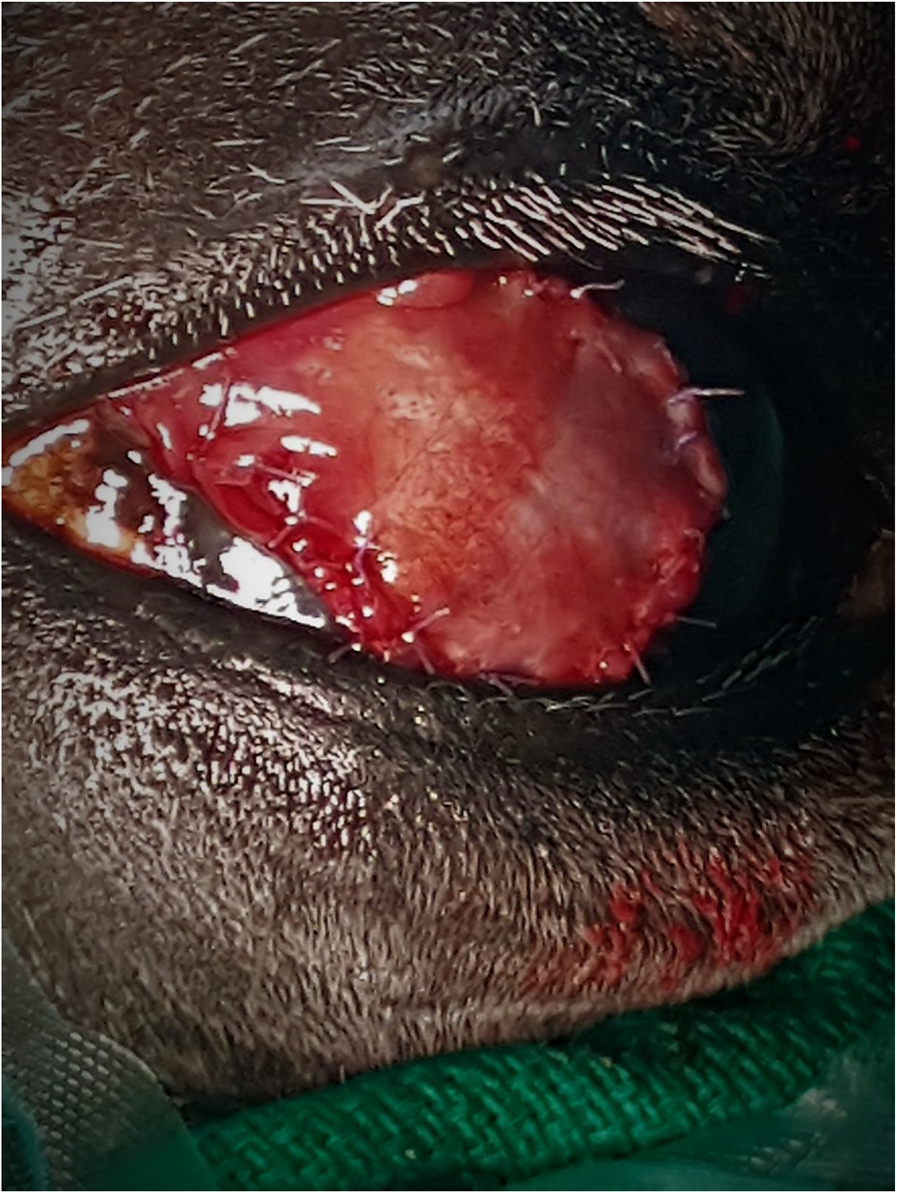
Intraoperative photograph illustrating the pedicle conjunctival flap created to cover the ulcer

The mare was sedated with xylazine (1.1 mg/kg, IV) and general anaesthesia was induced with ketamine (Ketamil, Troy laboratories PTY Limited, Glendenning, Australia) (2.2 mg/kg, IV) and diazepam (Diaz epam,Troy laboratories PTY Limited, Glendenning, Australia) (0.07 mg/kg, IV). The mare was positioned in left lateral recumbency and general anaesthesia was maintained using isoflurane in a circle line system. Subcutaneous auriculopalpebral and supraorbital nerve blocks were then performed using 0.5-1 mL 2% lidocaine hydrochloride. In addition, a retrobulbar nerve block was carried out using 15 mL 2% lidocaine hydrochloride. To further expose the globe, a lateral canthotomy was performed by making a full-thickness incision 5 mm in length, extending laterally from the lateral canthus. To ensure removal of all the necrotic stroma, a keratectomy was then carried out on the ulcerated cornea by dissection, using a No. 64 Beaver blade and Colibri corneal forceps (Eickemeyer Veterinary Inc.). The keratectomy was greater than 1/2 of the corneal thickness and was completed to leave a large (25 mm diameter) corneal defect as the bed for grafting. The infected tissues were removed and submitted for cytological examination. A large conjunctival pedicle graft was created using Westcott tenotomy scissors and Bishop-Harmon forceps (Eickemeyer Veterinary Inc.) to cover the ulcer. The conjunctiva was then sutured to the cornea using 6–0 polyglactin 910 in a simple interrupted suture pattern (Fig. 2). After completion of the surgery, the canthotomy was closed with two layers of 3–0 polyglactin 910, and a temporary tarsorrhaphy was performed by applying 4 horizontal mattress sutures using 3–0 polyamide 66, to provide additional corneal support. Anaesthetic recovery was uneventful. Cytology findings on the tissues collected surgically showed severe neutrophilic inflammation, along with epithelial cells. No intracellular bacteria or fungal elements were identified.

### Course of the condition

**Fig. 3:**
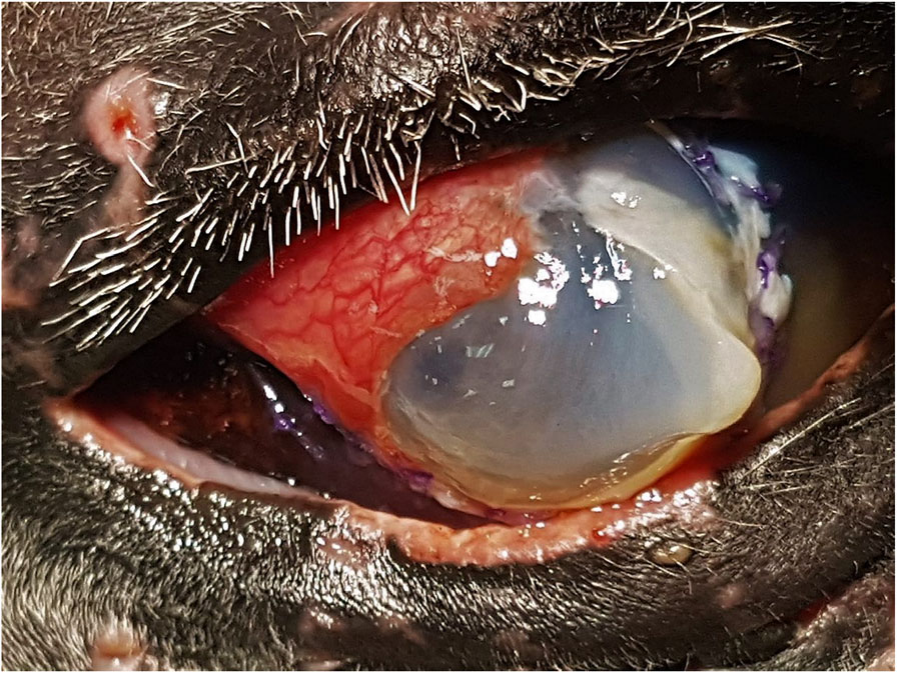
Appearance of the eye 7 days after the conjunctival flap. Note the graft had partially dehisced, the progressive corneal stromal liquefaction, and the presence of a central lateral focal descemetocele (bulging Descemet’s membrane)

The horse remained hospitalized and the previous medical treatment was continued for a further week. On Day 7 post-surgery, the temporal tarsorrhaphy was opened and the eye assessed. The graft had partially dehisced to leave a large central portion of the diseased cornea exposed. The corneal ulcer had enlarged (90% of the cornea diameter was now involved) and liquefaction (melting) was present with approximately 85% of corneal stroma loss, and a central lateral focal descemetocele of 5 mm diameter (Fig. 3). At this point, due to impending corneal perforation, enucleation or a second ocular surface reconstruction procedure was recommended with the sole purpose of preserving globe integrity. The owner declined enucleation, and a second grafting procedure was planned. By this time there was not enough remaining bulbar conjunctiva for a large pedicle graft and a tarsoconjuctival graft was considered unsuitable due to the large size of the corneal defect. Other commonly used corneal grafting materials such as amniotic membrane or extracellular matrix products were unavailable at the hospital. Hence, considering the advantages of a readily available, affordable, and easily harvested material, a segment of fascia lata from the ipsilateral hind limb of the horse was used as an autologous free graft with the principal aim of providing tectonic support during healing.

### Surgical technique and postoperative course

**Fig. 4:**
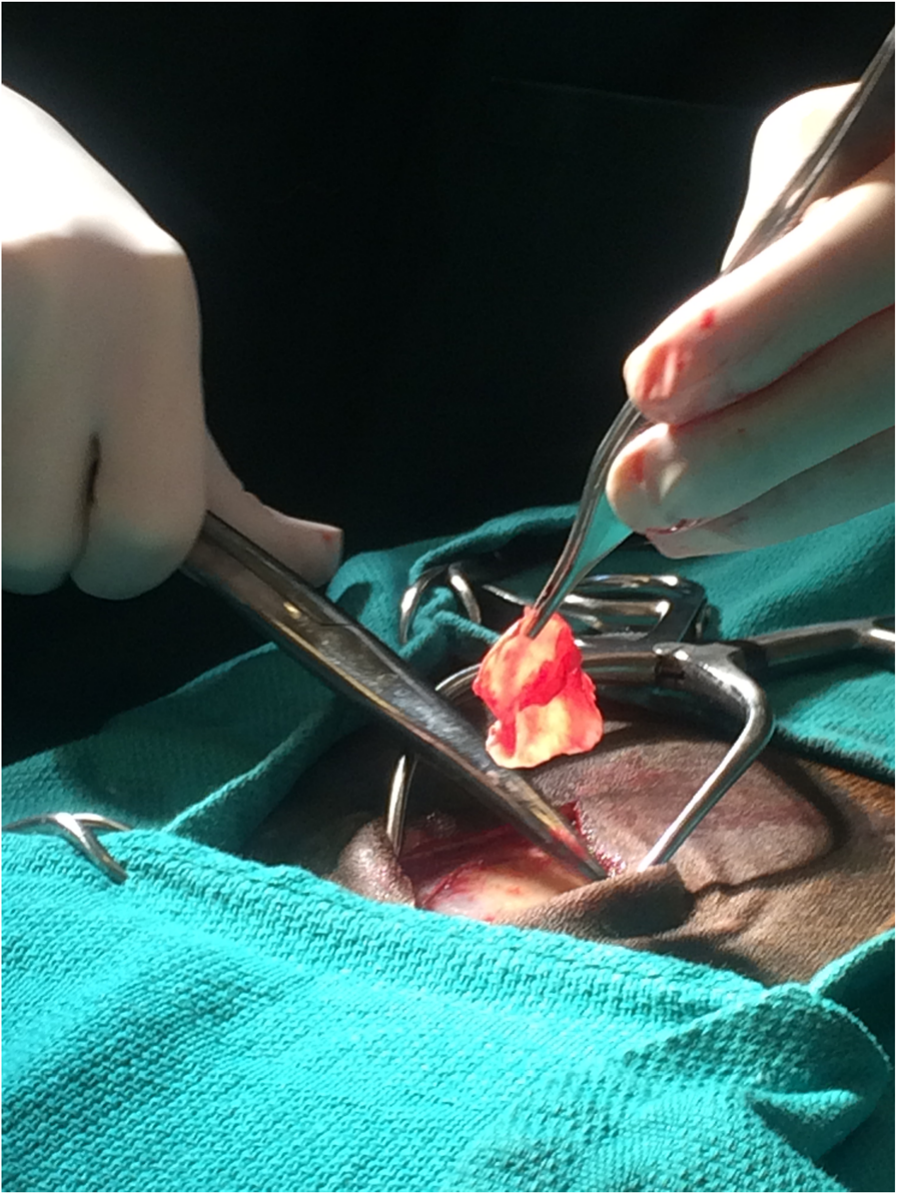
Intraoperative photograph illustrating the donor site at the time of fascia lata harvest in a pony

**Fig. 5:**
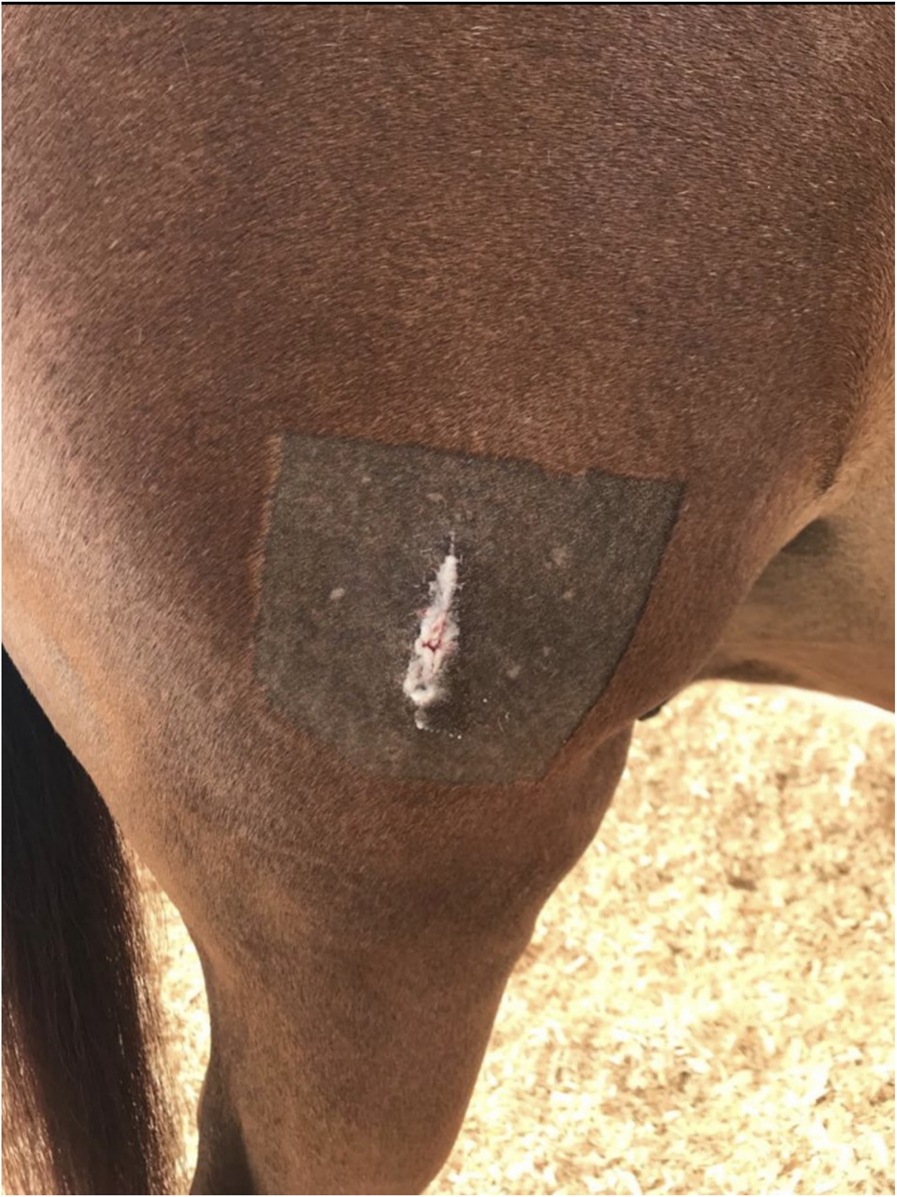
Photograph illustrating the donor site after two weeks of healing in pony

The mare was sedated with xylazine (1.1 mg/kg, IV) prior to induction, and anesthesia was induced using ketamine (2.2 mg/kg, IV) and diazepam (0.07 mg/kg, IV). The mare was positioned in left lateral recumbency and general anaesthesia was maintained using isoflurane (Isoflo, Aesica Queenborough Ltd., Kent, UK) in a circle line system. To harvest the facia graft, the skin over the lateral aspect of the right thigh was clipped and prepared for surgery. A 5 cm long proximodistal incision was made through the skin on the craniolateral aspect of the mid-thigh and the subcutaneous tissues were bluntly dissected to reveal the fascial sheath tissue surrounding the right thigh muscles (Figs. 4, 5). A circular section of fascia lata (2.5 cm × 2.5 cm) was excised and draped over a blood-soaked swab to attempt to maintain tissue integrity and prevent dehydration. The fascial defect was left open and the skin closed in two layers; subcutaneous tissues were closed using 2–0 Polyglactin 910 (Vicryl, Ethicon, Inc. Somerville, New Jersey, USA) in a simple continuous pattern, and 0 Polydioxanone (PDSII, Johnson & Johnson International, Leonardo Da Vincilaan, 15. Diegem. Belgium) was used in a simple interrupted pattern to close the skin. The previous lateral canthotomy was reopened after which, auriculopalpebral, supraorbital, and retrobulbar nerve blocks were performed using 2% lidocaine hydrochloride. The exposed area of necrotic corneal stroma where the conjunctival flap had dehisced was debrided and the malacic cornea removed using a No. 64 Beaver blade and Colibri corneal forceps. To ensure removal of all the necrotic stroma, a keratectomy greater than 3/4 of the corneal thickness was carried out by dissection to leave a 30 mm diameter corneal defect as the bed for grafting. The infected tissues were removed and submitted for cytology.

**Fig. 6:**
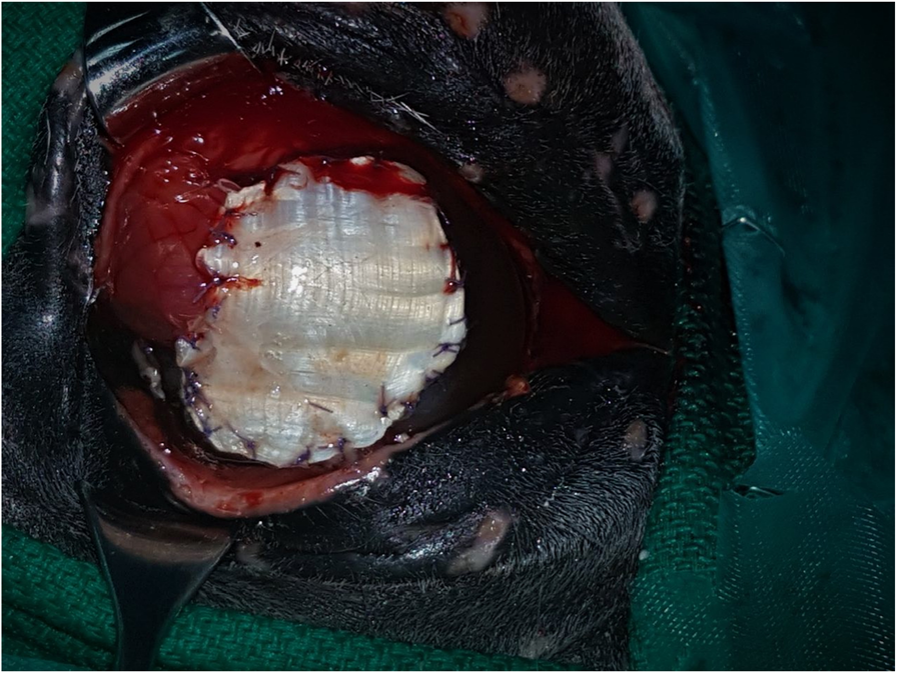
Intraoperative photograph illustrating the fascia lata graft covering the ulcer

The dorsal borders of the previous flap were well attached to the underlying cornea, so the remaining adherent conjunctiva was left in situ. The fascia lata flap was then used as an autologous free graft to cover the ulcer. It was positioned on the cornea, trimmed to size, and seated into the corneal defect. The graft was sutured into position using 6–0 polyglactin 910 in a simple interrupted pattern (Fig. 6). The lateral canthotomy was closed before a temporary tarsorrhaphy was performed to provide additional corneal support. Cytological examination of the collected tissues showed severe neutrophilic inflammation, along with epithelial cells but no intracellular bacteria or fungal elements were identified.

During the week following the second corneal grafting procedure the same treatment was continued except the intervals for flunixin meglumine and atropine administration were changed to q 24 h.

### Outcome and follow-up

**Fig. 7:**
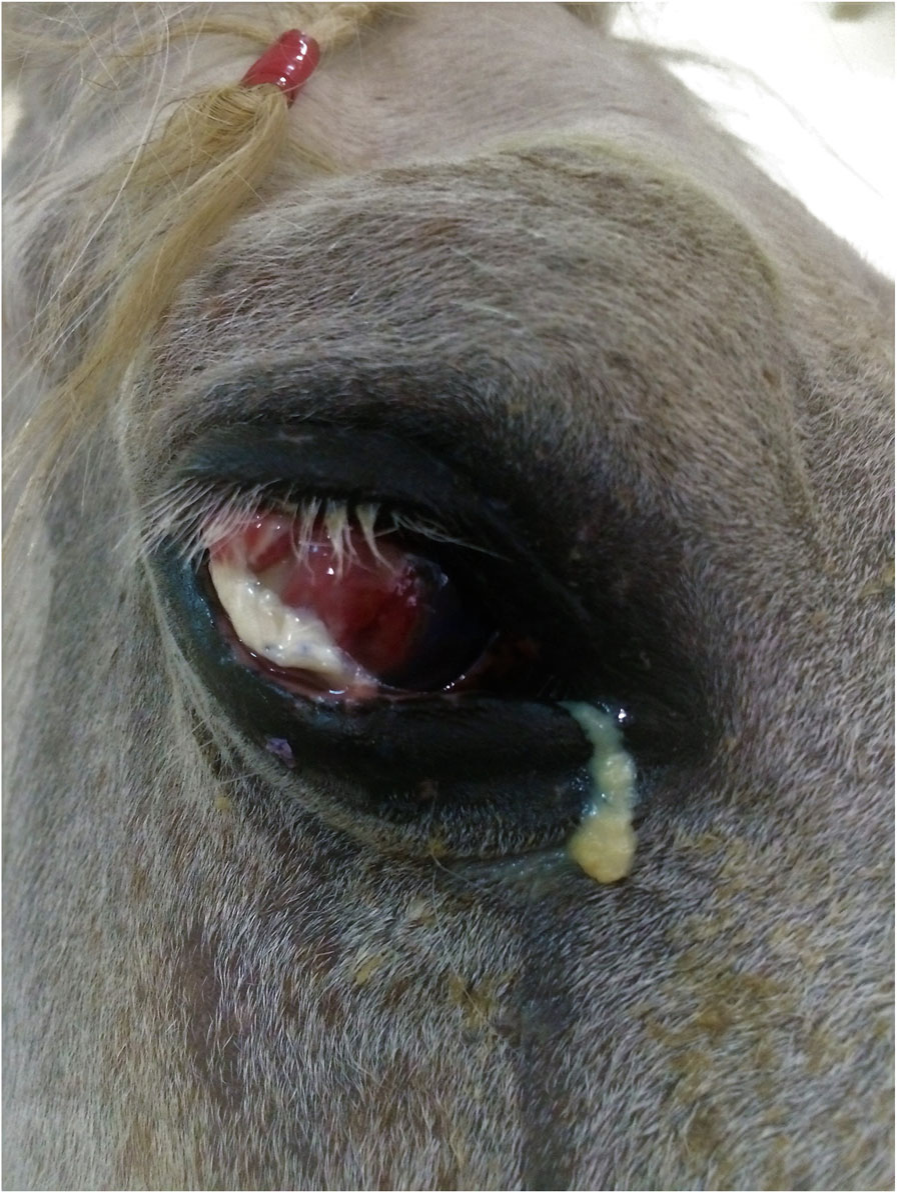
Illustrates the eye’s appearance 30 days after hospitalization (i.e. 16 days after corneal grafting). The fascial graft was degrading as a result of autolysis

**Fig. 8:**
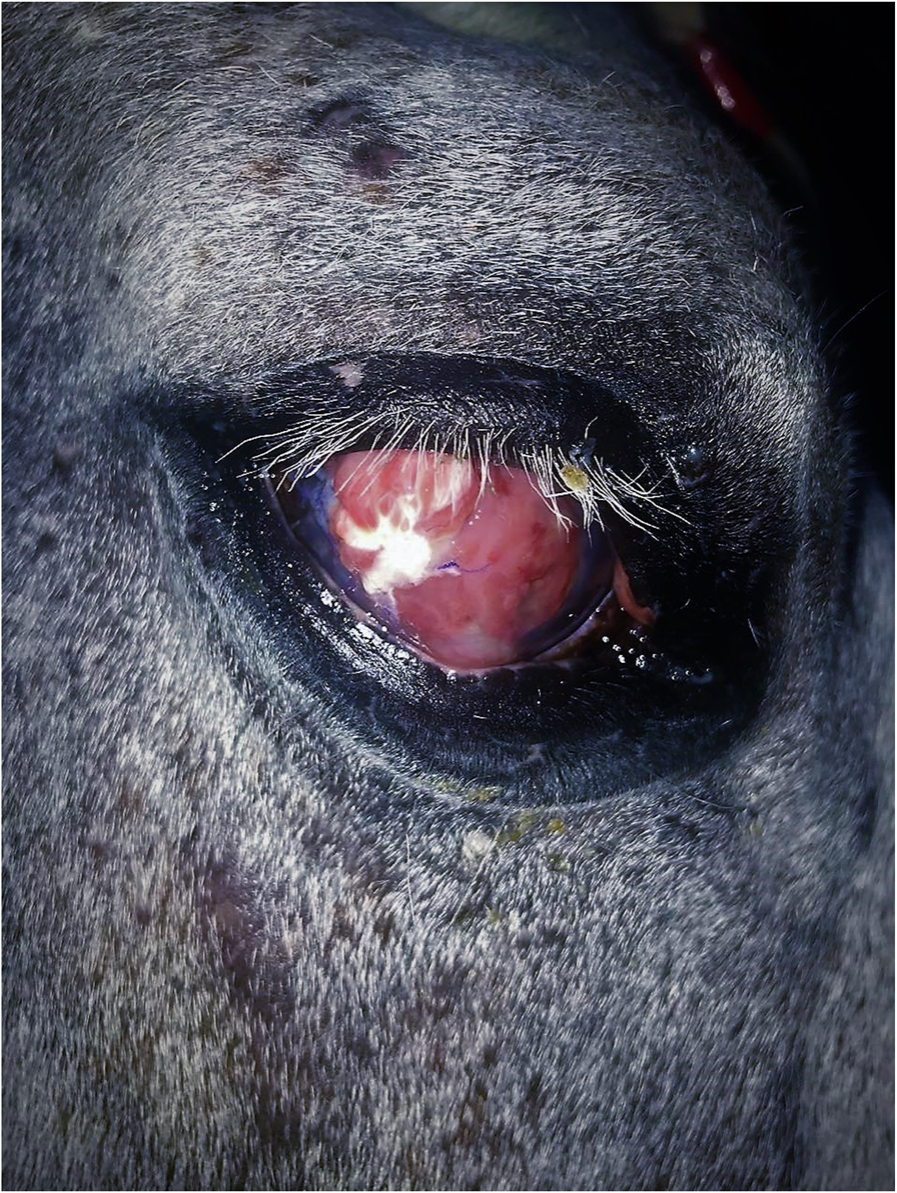
Illustrates the appearance of the eye at the time of discharge from the Hospital, 31 days after arrival and 17 days after fascia lata grafting. The graft has almost completely sloughed and the eye globe is stable without any evidence of corneal perforation

Seven days after the fascia lata graft was placed (Day 21 of hospitalization), the tarsorrhaphy was opened and the eye assessed. The graft was intact, and the horse seemed appreciably more comfortable showing only mild blepharospasm and epiphora. Fourteen days after the second graft (Day 28 of hospitalization), the fascia graft started to degrade while tissue repair was taking place over the corneal defect. The mare seemed comfortable with only mild blepharospasm and epiphora. Hence, the subpalpebral lavage system and the sutures from the donor site were removed, and the topical medications and flunixin meglumine were discontinued. Over the next 2 days, the graft continued to degrade and the eye remained comfortable (Fig. 7). On day 17 after the second surgery (Day 31 of hospitalization) the graft had completely sloughed off (Fig. 8) and the remaining sutures were removed. Ophthalmic examination demonstrated prominent superficial and deep peripheral corneal neovascularization extending to the grafted cornea and significant corneal scarring of the affected area with associated loss of transparency. The graft stained positively with fluorescein and the horse showed normal menace responses and dazzle reflex in just the temporal aspect of the eye. The presence of consensual and direct pupillary light responses in the injured eye were detected only via a limited view of the anterior chamber from the temporal aspect of the eye since no other corneal quadrants were accessible due to scarring. Similarly, only partial assessment of the anterior chamber was possible from the temporal aspect of the eye which revealed a mild inflammation with moderate flare. The posterior segment of the eye could not be examined due to corneal opacity. A Schirmer tear test was within normal limits [13] and no significant intraocular abnormalities were evident ultrasonographically [15]. The fascia donor site was also healing without complications. At this time, the horse was discharge with omeprazole as the only medication prescribed.

Follow-up examinations performed at 1, 4 and 12 months after the fascia lata grafting surgery consisted of STT, slit-lamp biomicroscopy, fluorescein staining, and ocular ultrasonography. No IOP measurements or indirect ophthalmoscopy were available at the time.

At 1 month post-surgery, the mare was comfortable and the appearance of the right eye continued to improve. Ophthalmic examination demonstrated that corneal vascularisation was still present and fluorescein staining showed a 10 mm diameter epithelial defect over the grafted area. Some vision was evident from normal menace responses, dazzle reflex and the presence of consensual and direct pupillary light responses (PLR) in the injured eye (PLR were detected via a limited view of the anterior chamber from the temporal aspect of the eye; no other corneal quadrants were accessible due to scarring). Assessment of the anterior chamber from the temporal aspect of the eye revealed minimal flare. A Schirmer tear test was within normal limits [13] and no significant intraocular ultrasonographic abnormalities were present. The fascia lata donor site had healed with minimal scarring, with no pain or perceptible functional impairment.

At the second recheck examination, 4 months postoperatively, there was evidence that the horse still had some vision in the affected eye, as demonstrated by a positive dazzle reflex and consistent menace response on the temporal side. Fluorescein staining was negative and the periphery of the cornea showed minimal and regressing vascularisation. Ocular ultrasonography was within normal limits. Apart from the poor visual outcome due to central opaque corneal scarring, the results of the ophthalmic examination were within normal limits, and without signs of ocular discomfort.

**Fig. 9:**
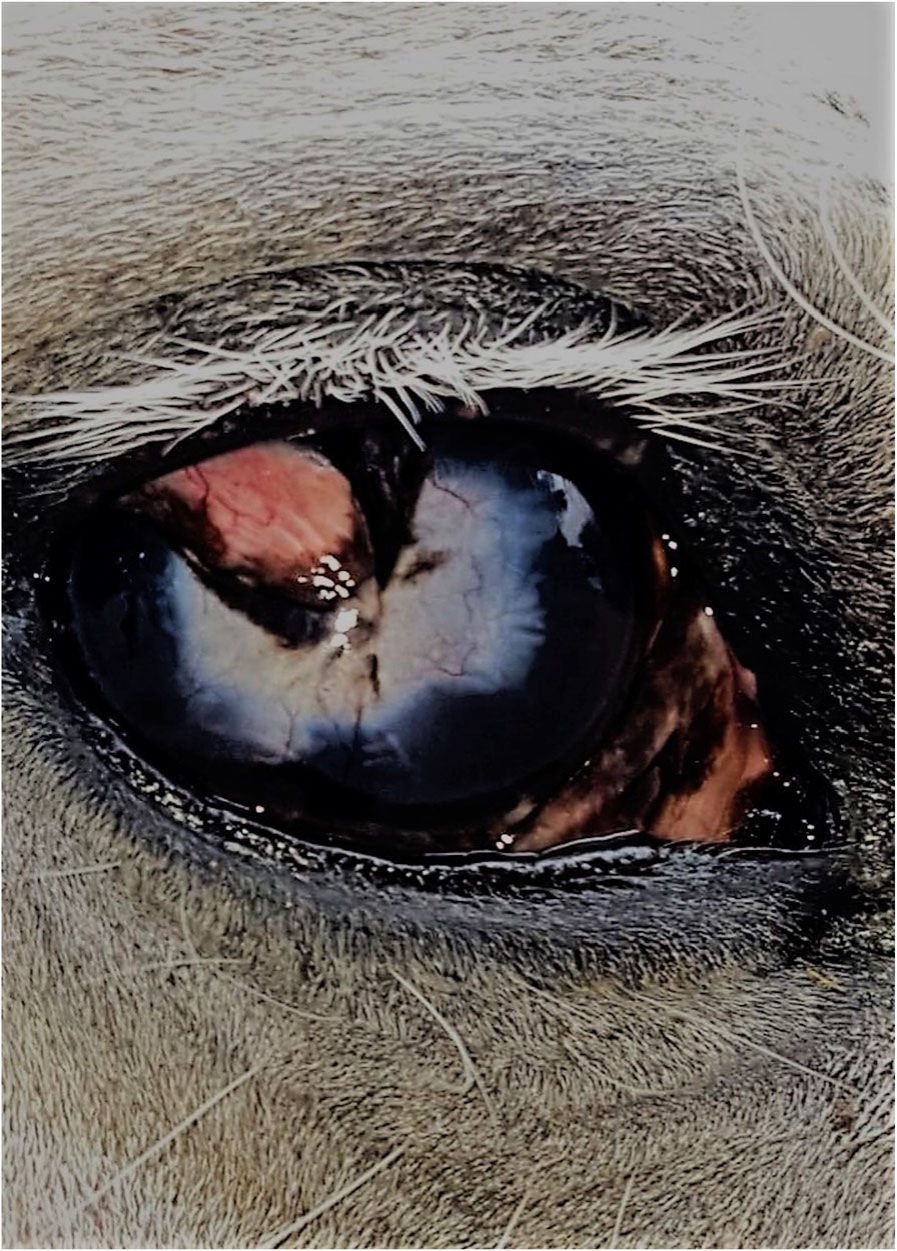
The appearance of the eye one year after discharge. Note the central corneal fibrosis and the partially pigmented conjunctival flap that was left after dehiscence of the first conjunctival pedicle graft

By 12 months postoperatively, results of the ophthalmic examination, including ocular ultrasonography, were within normal limits. The mare remained comfortable and the eye partially visual due to the central opaque corneal scarring. The owner was pleased to have saved the globe, despite this low visual outcome (Fig. 9).

## Discussion/Conclusions

This report documents a new technique for repair of complex defects of the equine cornea. Treatment of collagenolytic keratitis is either medical, surgical or both. Initial medical therapy for infectious keratitis has been routinely based on treatment of the cornea with topical antimicrobials, cycloplegics to dilate the pupil and reduce ciliary spasm, systemic anti-prostaglandin synthetase inhibitors for pain relief and anticollagenase medications all applied topically [2]. In cases of primary fungal keratitis, beside prophylactic antibiotic medication, medical therapy must be directed against the fungus and a topical, broad-spectrum, antifungal agent is appropriate while awaiting culture and sensitivity results. Topical voriconazole is an excellent first choice of therapy because it can effectively penetrate the cornea, reach detectable concentrations in the aqueous humor and it has broad antifungal spectrum [16].

Additionally, intra-stromal and subconjunctival injection of voriconazole solution under standing sedation have been recommended [17, 18]. Corneal cross-linking with photoactivated riboflavin is a relatively new medical approach which is being used increasingly by human and veterinary ophthalmologists to manage severe infectious keratitis associated with corneal melting [19]. However, significant differences exist between different species with regard to the penetration depth of corneal cross-linking, and the current protocols for the horse still need refinement [20].

Intraocular inflammation with associated anterior uveitis, or iridocyclitis, is always present in cases of equine keratomalacia caused by infection. In particular with fungal keratitis, it can escalate in intensity following death of hyphae after antifungal therapy is initiated [14]. In the present case we used flunixin meglumine, a prostaglandin synthetase inhibitor, for systemic treatment of iridocyclitis. In addition, the mydriatic agent, atropine, was added to the topical therapy for pain control and prevention of synechiae [14].

When medical therapy is unsuccessful, progressive melting and necrosis of the corneal stroma may lead to descemetocele formation and perforation of the cornea. Therefore, any corneal defect deep enough to threaten corneal integrity warrants surgical repair by corneal reconstruction/grafting [2]. The goal in ocular surface reconstruction is to preserve the integrity of the globe, provide tectonic support, optimize the visual outcome, and minimize corneal scar formation [2, 4]. Loss of optical clarity occurs with all biomaterials, whereas minimal scarring occurs when the cornea heals by medical treatment alone, without corneal grafting [11].

Corneal transplantations, including fresh or frozen corneal allografts and autografts, have been documented in the veterinary literature and have met with some success. However in cases of infections and collagenolytic keratitis the incidence of graft rejection is high [21]. Although grafts as large as 25 mm diameter have been reported, the most commonly used sizes which have the best record of surgical success are 6–8 mm in diameter. Fresh, homologous tissue for corneal transplantation is indicated where minimal corneal opacity is important. However, it requires the availability of fresh donor tissue at the time needed, which makes such fresh corneal grafts a rare occurrence in veterinary ophthalmology [3]. Frozen, homologous grafts are more commonly used to provide tectonic support but their rejection, manifested clinically as vascularisation of the graft, results in greater permanent fibrotic scarring at the graft site and is reported as a frequent complication [9, 21]. In the present case and due to the extent of the corneal lesion, a corneal transplantation graft was not considered to be the optimum therapy in the first instance.

Corneoconjunctival transpositions are autologous partial-thickness corneal grafts in which peripheral cornea is grafted into the axial cornea, so as to maintain its conjunctival vascular attachments. The procedure is indicated for axial stromal defects of < 30% of the corneal diameter [22, 23]. In the present case, the extent of the defect was estimated to be 90% of the cornea.

A commonly performed grafting procedure in horses is the conjunctival pedicle graft in which the base of the graft retains a connection to the bulbar conjunctiva [2]. Conjunctiva lends satisfactory structural integrity to a compromised cornea and it provides blood vessels to an otherwise avascular region. However, the conjunctival tissue remains at the site of graft, which results in a permanent corneal opacity that impairs vision when lesions are extensive or graft resection is impractical [3]. Additionally, as in the present case, due to the extension and severity of the melting process, the structural support of the conjunctiva was considered inferior to the structural support provided by fascia lata.

Other biomaterials commonly used for corneal reconstruction in veterinary patients include amniotic membrane, porcine small intestine submucosa, porcine urinary bladder extracellular matrix, and bovine pericardium [3, 5, 6, 10].

Commercially available porcine urinary bladder extracellular matrix and small intestine submucosa grafts act as scaffolds that first promote cell proliferation and regeneration, then degrade as local tissues replace the graft [3]. An advantage of porcine small intestine submucosa is its complete remodeling and incorporation into the host cornea, thereby minimising scarring [3]. In veterinary ophthalmology successful surgical repair has been reported in several melting ulcer cases with good corneal transparency, preservation of corneal integrity and the maintenance of vision [24]. In the present case, the commercially available discs of these two porcine products (15 mm in diameter) were considered too small for the lesion.

Commercially available bovine pericardium grafts offer a promising option for surgical reconstruction of the cornea to manage deep melting corneal ulcers [10]. This biomaterial is commercially available and it has been used successfully in several veterinary ophthalmology cases [10]. However, further investigations are required to assess its efficacy for treatment of infected corneal ulcers [10].

Transplants of amniotic membrane have been described as an alternative to conjunctival pedicle grafting which may provide some tectonic support as well as improved postoperative optical clarity resulting from less fibrotic scarring, and less iatrogenic trauma when compared to conjunctival pedicles [3, 4]. In addition, amnion possesses anti-inflammatory properties, it contains growth factors and it is anti-fibrotic [4, 25]. However, harvesting and preparating amniotic tissue can be challenging and time-consuming, and commercially available amniotic membrane for corneal grafting may not be strong, or large enough to support extensive, severe malacic ulcers. These products may also not be widely available. Harvested horse amniotic membrane was unavailable at the hospital where treatment was undertaken, and the commercially available discs or plates were not available at the time.

In the present case, a pedicle conjunctival graft was carried out during the first surgical intervention. Unfortunately, dehiscence was observed at removal of the tarsorrhaphy on Day 7 post operatively. Dehiscence was attributed to the aggressive nature of the ulcerative process and at that time, enucleation of the eye or a second ocular salvage procedure were given as surgical options. The client elected for the horse to have a second ocular surface reconstruction and so the decision was made to harvest a segment of fascia lata from the ipsilateral hind limb and use this as an autologous graft, primarily due to a lack of other biomaterials at the hospital but also due to its great strength, ready availability, ease of harvesting, and relatively low cost.

Initial and postoperative medical treatment included systemic administration of flunixin meglumine while local therapy included voriconazole drops, ciprofloxacin drops, atropine drops, and autogenous serum instilled into the lavage system. Topical ciprofloxacin was empirically chosen based on the hospital general infectious keratitis antibiotic sensitivity patterns. However, fluoroquinolones have been shown to have severe cytopathological effects on corneal epithelium that could have impaired further healing [10]. Systemic antibiotics are recommended and frequently used for deep melting corneal ulcers [10, 24]. However, no systemic antibiotics were given to the present horse due to the lack of any bacterial growth on culture.

Two weeks after the fascia lata graft, the mare seemed comfortable with mild blepharospasm and epiphora so at that point, topical medications and systemic flunixin meglumine were discontinued. In hindsight, continuation of the topical anti-inflammatory therapy would have been beneficial to minimise residual corneal vessels and inflammatory infiltration [10].

Autologous grafts are free of antigenicity problems and fascia lata, in particular, is an excellent scaffolding graft that functions as an inductive template to help tissue regeneration [26]. This biologically uniform mesh of elastic fibres has a proven track record in human surgery where it is used successfully for many indications, including tympanoplasties (tympanic membrane perforation repairs) [27], abdominal fascia defect repair (e.g. enterocutaneous fistula, or wound dehiscence with mesh exposure) [28], plastic procedures (e.g. cleft lip deformities, reconstruction of deep ulcers in diabetic patients, or rhinoplasty in thin-skinned patients) [29–31], orthopedic procedures (e.g. anterior cruciate ligament reconstruction, temporomandibular joint ankyloses, or rotator cuff augmentation surgery) [26, 32, 33], urologic and gynecologic procedures (e.g. complex penile defect repair, surgical management of Peyronie’s disease) [34, 35] and also several ophthalmic techniques (e.g. congenital blepharoptosis correction, or traumatic eye globe luxation repair) [36, 37]. Conversely, fascia lata grafting in veterinary surgery has seldom been reported, with only one case described in a dog that sustained a traumatic scleral rupture. The scleral defect was repaired using autogenous fascia lata graft and then the conjunctiva was sutured over the graft. In that case, the graft gave excellent tectonic support and it resulted in a comfortable and functional eye at 3 months postsurgery [12].

In the present case, the harvested fascial material proved to be substantially thicker and stronger than the traditional conjunctival flap, or commonly used amnion, and it was therefore expected to show greater tectonic properties. However, while the fascia graft failed to provide tissue integration due to insufficient vascularity, it did endure over time and it gave additional physical support to protect the cornea during the first weeks after surgery while tissue repair was taking place [26]. The fact that the tissue degrades as local tissues replace the graft, such that no foreign material remains, may also be associated with a reduction in fibrotic scarring and better long-term transparency.

It is recognised that there were problems with certain aspects of this case report. The follow-up of clinical cases in veterinary practice is often limited by financial and practical constraints. The fact that no veterinary ophthalmologist was involved in the case was due to accessibility, and potentially impacted the quality of the initial and follow-up ocular examinations. Intraocular pressure was not monitored in this mare due to the unavailability of tonometry. The onset of glaucoma is a known serious complication of inflammatory ocular disease and should have been monitored throughout the horse’s recovery [38]. Surgically, the use of 6–0 polyglactin 910 as the suture material for corneal repair was due to its availability rather than choice. Such suture material is generally considered too thick for corneal repair especially where there is considerable stromal loss. The recommended suture material size for corneal grafting is 8–0 or 9–0 [3, 10, 11]. Additionally, the fact that a small portion of the initial conjunctival flap remained in place, could have contributed in part to the successful healing process. Concerning the suboptimal visual outcome, the remaining conjunctival pedicle graft likely contributed to this as well.

Although the fascia lata corneal grafting technique is considered effective to protect and support a severely malacic cornea, additional cases and longer follow-up times are needed to further justify its use, together with further refinement of the technique, and a better description of its potential short- and long- term complications.

In summary, fascia lata appears to be an effective and affordable autografting material, with minimal donor site morbidity, that may be considered for tectonic support of severe malacic or other complex corneal ulcers. This may be particularly relevant in cases where other types of grafting biomaterials are not available.

## Data Availability

Not applicable.

## Abbreviations

STT: Schirmer tear test
PLR: Pupillary light responses
